# [6-(Furan-2-yl)-7-nitro-2,3,4,6,7,8-hexa­hydro-1*H*-pyrido[1,2-*a*]pyrimidin-9-yl](phen­yl)methanone

**DOI:** 10.1107/S160053681301386X

**Published:** 2013-05-31

**Authors:** Wen-Feng Zhang, Jian-Hui Xia, Zhao-Hui Xu, Li-Ben Wang, Chu-Yi Yu

**Affiliations:** a College of Chemistry and Chemical Engineering, Jiang Xi Normal University, Nanchang, Jiang Xi 330022, People’s Republic of China; b Beijing National Laboratory for Molecular Science (BNLMS), CAS Key Laboratory of Molecular Recognition and Function, Institute of Chemistry, Chinese Academy of Sciences, Beijing 100190, People’s Republic of China

## Abstract

The asymmetric unit of the title compound, C_19_H_19_N_3_O_4_, contains two mol­ecules with very few conformational differences; a C atom in the pyrimidine ring in one of the mol­ecules is disordered in a 0.688 (15):0.312 (15) ratio. In both mol­ecules, the fused pyridine and pyrimidine rings adopt half-chair conformations. The dihedral angles between the furan and benzene rings are 81.00 (13) and 84.99 (10)° in the two mol­ecules. The mol­ecular structure is consolidated by intra­molecular N—H⋯O hydrogen bonding. In the crystal, C—H⋯O hydrogen bonds connect the molecules into a three-dimensional network.

## Related literature

For background to heterocyclic ketene aminals, see: Yaqub *et al.* (2008 ▶); Huang & Wang (1994 ▶); Yu *et al.* (2006 ▶); For the crystal structure of a related compound, see: Yu *et al.* (2007 ▶).
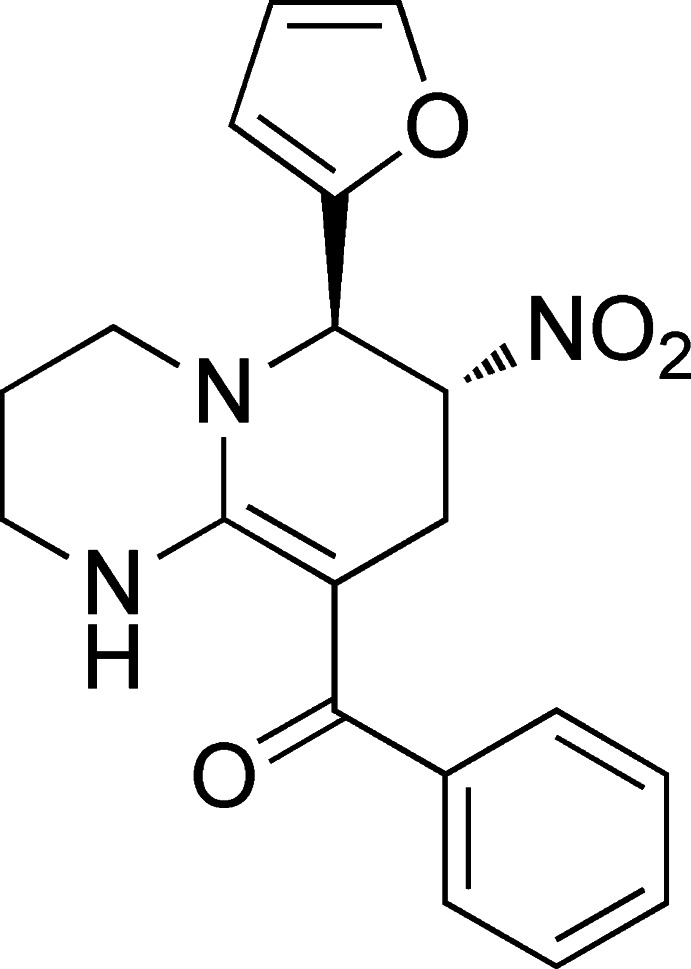


## Experimental

### 

#### Crystal data

C_19_H_19_N_3_O_4_
*M* *_r_* = 353.37
Monoclinic, 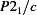

*a* = 13.506 (3) Å
*b* = 17.675 (4) Å
*c* = 14.755 (3) Å
β = 105.12 (3)°
*V* = 3400.3 (12) Å^3^
*Z* = 8
Mo *K*α radiation
μ = 0.10 mm^−1^
*T* = 173 K
0.44 × 0.43 × 0.37 mm

#### Data collection

Rigaku R-AXIS RAPID IP area-detector diffractometer
Absorption correction: multi-scan (*ABSCOR*; Higashi, 1995 ▶) *T*_min_ = 0.958, *T*_max_ = 0.964
11765 measured reflections
5990 independent reflections
4405 reflections with *I* > 2σ(*I*)
*R*_int_ = 0.024

#### Refinement

*R*[*F* ^2^ > 2σ(*F* ^2^)] = 0.049
*wR*(*F* ^2^) = 0.126
*S* = 1.02
5990 reflections
479 parameters
40 restraints
H-atom parameters constrained
Δρ_max_ = 0.59 e Å^−3^
Δρ_min_ = −0.26 e Å^−3^

### 

Data collection: *RAPID-AUTO* (Rigaku, 2001 ▶); cell refinement: *RAPID-AUTO*; data reduction: *RAPID-AUTO*; program(s) used to solve structure: *SHELXS97* (Sheldrick, 2008 ▶); program(s) used to refine structure: *SHELXL97* (Sheldrick, 2008 ▶); molecular graphics: *SHELXTL* (Sheldrick, 2008 ▶); software used to prepare material for publication: *SHELX97*.

## Supplementary Material

Crystal structure: contains datablock(s) I, global. DOI: 10.1107/S160053681301386X/pv2626sup1.cif

Structure factors: contains datablock(s) I. DOI: 10.1107/S160053681301386X/pv2626Isup2.hkl

Supplementary material file. DOI: 10.1107/S160053681301386X/pv2626Isup3.cml

Additional supplementary materials:  crystallographic information; 3D view; checkCIF report

**Fig. 1.**
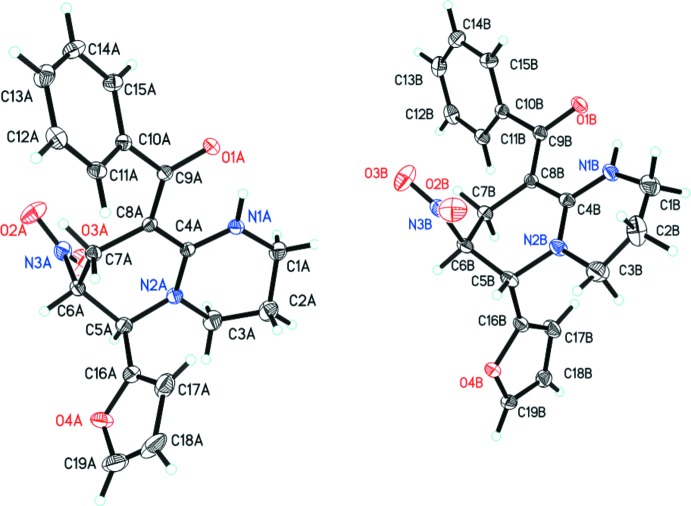
The molecular structure of the title compound showing both molecules in the asymmetric unit. Displacement ellipsoids are drawn at the 30% probability level and the smaller fraction (C2D) of the disordered atom has been ignored.

Enhanced figure: interactive version of Fig. 1

**Fig. 2.**
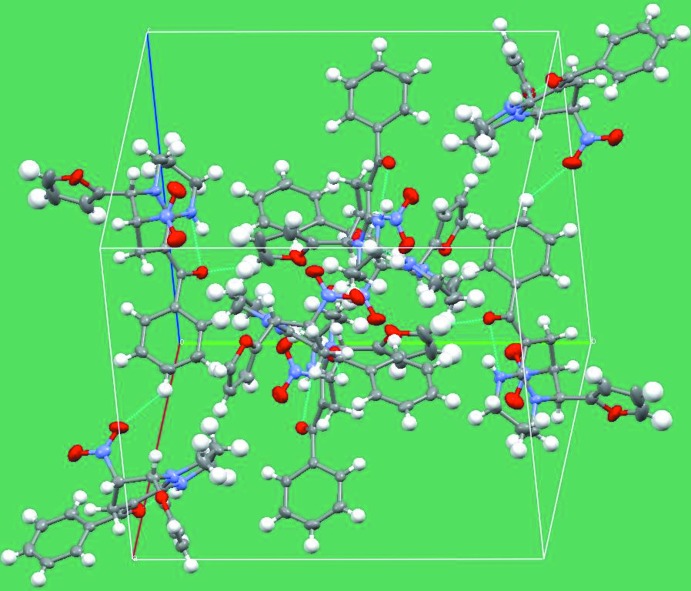
The crystal packing for title compound, with intramolecular hydrogen bonding N—H···O and intermolecular C—H···O hydrogen bonding interactions shown as dashed lines.

Enhanced figure: interactive version of Fig. 2

## Figures and Tables

**Table 1:** Hydrogen-bond geometry (Å, °)

*D*—H⋯*A*	*D*—H	H⋯*A*	*D*⋯*A*	*D*—H⋯*A*
N1*A*—H1*AA*⋯O1*A*	0.88	1.84	2.553 (2)	137
N1*B*—H1*BA*⋯O1*B*	0.88	1.85	2.556 (3)	136
C6*A*—H6*AA*⋯O1*B*	1.00	2.36	3.103 (3)	130
C7*A*—H7*AB*⋯O2*A*	0.99	2.44	2.790 (3)	100

## supplementary crystallographic information

### Comment

Heterocyclic ketene aminals (HKAs), by high caliber of distinctive electronic feature, have been widely used as flexible synthons for the construction of a variety of fused-ring polycyclic heterocycles (Huang & Wang, 1994; Yu *et al.*, 2006; Yaqub *et al.*, 2008) and therefore highly noteworthy in contemporary organic synthesis.

The asymmetric unit of the title compound, is composed of two molecules with very little conformational differences; a C-atom in the pyrimidine ring in one of the molecules is disordered in 0.688 (15):0.312 (15) ratio. The bond distances and angles in both molecules of the title compound (Fig. 1) agree very well with the corresponding bond distances and angles reported in a closely related compound (Yu *et al.*, 2007). The fused pyridyl (N2*A*/C4*A*—C8*A*), (N2*B*/C4*B*—C8*B*) and pyrimidyl (N1*A*/N2*A*/C1*A*—C3*A*/C4*A*), (N1*B*/N2*B*C1*B*—C3*B*/C4*B*) rings adopt half-chair conformations. The atoms C2A, C6A, C6B, C2B and C2D lie 0.647 (4), 0.611 (3), 0.614 (3), -0.295 (10) and 0.523 (10) Å, respectively, out of the planes formed by the remaining ring atoms. The molecular packing in the crystal structure is consolidated by intermolecular C—H···O hydrogen bonding (Table 1 & Fig. 2).

### Experimental

(*E*)-3-(furan-2-yl)-2-nitroallyl acetate (211 mg, 1 mmol) and 1-phenyl-2- (tetrahydropyrimidin-2(1*H*)-ylidene)ethanone (202 mg, 1 mmol) were stirred in 20 ml of dichloromethane at 273 K for 1 h. Then the temperature was allowed to rise up to the room temperature and stirred for another 6 h. The solvent was removed *in vacuo* and the residue was purified by flash column chromatography (silica gel, petroleum ether/EtOAc 1:1) to give the title compound as a light yellow solid. The single crystals of the title compound were grown in dichloromethane - petroleum ether (1:5) system at room temperature by slow evaporation. Yield: 75% (264.8 mg), m.p. 457–458 K.

### Refinement

All H atoms were positioned geometrically and refined using a riding model with distances C—H = 0.95, 0.99 or 1.00 Å for aryl, methylene and methine H-atoms, respectively, and N—H = 0.88 Å. The H-atoms are constrained to ride on their parent atoms, with *U*_iso_(H) = 1.2 times *U*_eq_(C/N). The atom C2*B* and C2*D* of molecule B are disordered with sire occupancy factors 0.688 (15) and 0.312 (15), respectively; the commands ISOR and SADI were used to model the disorder.

### Crystal data

**Table:**

C_19_H_19_N_3_O_4_	*F*(000) = 1488
*M**_r_* = 353.37	*D*_x_ = 1.381 Mg m^−^^3^
Monoclinic, *P*2_1_/*c*	Mo *K*α radiation, λ = 0.71073 Å
Hall symbol: -P 2ybc	Cell parameters from 55355 reflections
*a* = 13.506 (3) Å	θ = 2.2–27.5°
*b* = 17.675 (4) Å	µ = 0.10 mm^−^^1^
*c* = 14.755 (3) Å	*T* = 173 K
β = 105.12 (3)°	Block, yellow
*V* = 3400.3 (12) Å^3^	0.44 × 0.43 × 0.37 mm
*Z* = 8	

### Data collection

**Table:**

Rigaku R-AXIS RAPID IP area-detector diffractometer	5990 independent reflections
Radiation source: rotating anode	4405 reflections with *I* > 2σ(*I*)
Graphite monochromator	*R*_int_ = 0.024
ω scans at fixed χ = 45°	θ_max_ = 25.0°, θ_min_ = 2.2°
Absorption correction: multi-scan (*ABSCOR*; Higashi, 1995)	*h* = −16→16
*T*_min_ = 0.958, *T*_max_ = 0.964	*k* = −20→21
11765 measured reflections	*l* = −17→17

### Refinement

**Table:**

Refinement on *F*^2^	Primary atom site location: structure-invariant direct methods
Least-squares matrix: full	Secondary atom site location: difference Fourier map
*R*[*F*^2^ > 2σ(*F*^2^)] = 0.049	Hydrogen site location: inferred from neighbouring sites
*wR*(*F*^2^) = 0.126	H-atom parameters constrained
*S* = 1.02	*w* = 1/[σ^2^(*F*_o_^2^) + (0.0698*P*)^2^] where *P* = (*F*_o_^2^ + 2*F*_c_^2^)/3
5990 reflections	(Δ/σ)_max_ < 0.001
479 parameters	Δρ_max_ = 0.59 e Å^−^^3^
40 restraints	Δρ_min_ = −0.26 e Å^−^^3^

### Special details

**Table:**

Geometry. All e.s.d.'s (except the e.s.d. in the dihedral angle between two l.s. planes) are estimated using the full covariance matrix. The cell e.s.d.'s are taken into account individually in the estimation of e.s.d.'s in distances, angles and torsion angles; correlations between e.s.d.'s in cell parameters are only used when they are defined by crystal symmetry. An approximate (isotropic) treatment of cell e.s.d.'s is used for estimating e.s.d.'s involving l.s. planes.
Refinement. Refinement of *F*^2^ against ALL reflections. The weighted *R*-factor *wR* and goodness of fit *S* are based on *F*^2^, conventional *R*-factors *R* are based on *F*, with *F* set to zero for negative *F*^2^. The threshold expression of *F*^2^ > σ(*F*^2^) is used only for calculating *R*-factors(gt) *etc*. and is not relevant to the choice of reflections for refinement. *R*-factors based on *F*^2^ are statistically about twice as large as those based on *F*, and *R*- factors based on ALL data will be even larger.

### Fractional atomic coordinates and isotropic or equivalent isotropic displacement parameters (Å^2^)

**Table:**

	*x*	*y*	*z*	*U*_iso_*/*U*_eq_	Occ. (<1)
O1A	0.55143 (11)	0.64789 (9)	0.96144 (10)	0.0292 (4)	
O1B	0.20201 (12)	0.49311 (9)	0.48209 (10)	0.0348 (4)	
O2A	0.26198 (16)	0.64416 (12)	0.65380 (14)	0.0602 (6)	
O2B	0.04702 (15)	0.49215 (12)	0.10899 (13)	0.0542 (5)	
O3A	0.36728 (16)	0.63894 (12)	0.56801 (15)	0.0619 (6)	
O3B	0.00216 (17)	0.38763 (12)	0.16041 (15)	0.0612 (6)	
O4B	−0.24369 (11)	0.62772 (9)	0.13117 (10)	0.0287 (4)	
N1A	0.63716 (13)	0.62164 (11)	0.82954 (13)	0.0298 (4)	
H1AA	0.6354	0.6458	0.8813	0.036*	
N1B	0.17129 (14)	0.60783 (11)	0.37285 (13)	0.0324 (5)	
H1BA	0.2053	0.5863	0.4256	0.039*	
N2A	0.54886 (13)	0.55555 (11)	0.69830 (12)	0.0277 (4)	
N2B	0.02541 (14)	0.61336 (11)	0.25015 (12)	0.0322 (5)	
N3B	−0.00221 (15)	0.45606 (13)	0.15228 (13)	0.0373 (5)	
N3A	0.32970 (16)	0.61309 (12)	0.62734 (13)	0.0359 (5)	
C1A	0.73214 (18)	0.62515 (15)	0.80068 (18)	0.0383 (6)	
H1AB	0.7915	0.6205	0.8563	0.046*	
H1AC	0.7370	0.6744	0.7703	0.046*	
C1B	0.2157 (2)	0.67467 (16)	0.3425 (2)	0.0496 (7)	
H1B1	0.2212	0.7144	0.3908	0.060*	
H1B2	0.2862	0.6622	0.3394	0.060*	
C3B	0.0542 (2)	0.68725 (16)	0.2204 (2)	0.0514 (8)	
H3B1	0.0364	0.6888	0.1509	0.062*	
H3B2	0.0136	0.7269	0.2417	0.062*	
C2B	0.1630 (9)	0.7045 (8)	0.2566 (8)	0.052 (4)	0.312 (15)
H2B1	0.1983	0.6874	0.2093	0.063*	0.312 (15)
H2B2	0.1700	0.7602	0.2609	0.063*	0.312 (15)
C2D	0.1329 (4)	0.7263 (3)	0.2906 (4)	0.0385 (15)	0.688 (15)
H2DA	0.1008	0.7512	0.3360	0.046*	0.688 (15)
H2DB	0.1635	0.7663	0.2595	0.046*	0.688 (15)
C2A	0.73418 (19)	0.56157 (17)	0.73265 (18)	0.0451 (7)	
H2AA	0.7922	0.5691	0.7042	0.054*	
H2AB	0.7443	0.5127	0.7666	0.054*	
C3A	0.63701 (18)	0.55976 (17)	0.65830 (17)	0.0416 (6)	
H3AA	0.6315	0.6059	0.6192	0.050*	
H3AB	0.6367	0.5153	0.6173	0.050*	
C4A	0.55271 (16)	0.58496 (12)	0.78425 (14)	0.0234 (5)	
C4B	0.08267 (16)	0.57667 (13)	0.32670 (14)	0.0251 (5)	
C5A	0.46227 (17)	0.51183 (13)	0.64412 (14)	0.0287 (5)	
H5AA	0.4545	0.5232	0.5762	0.034*	
C5B	−0.06863 (16)	0.58213 (14)	0.18796 (14)	0.0299 (5)	
H5BA	−0.0684	0.5933	0.1216	0.036*	
C6A	0.36290 (16)	0.53476 (13)	0.66721 (14)	0.0268 (5)	
H6AA	0.3083	0.4976	0.6372	0.032*	
C6B	−0.07479 (16)	0.49688 (13)	0.19890 (14)	0.0283 (5)	
H6BA	−0.1464	0.4804	0.1677	0.034*	
C7B	−0.05028 (16)	0.47362 (13)	0.30167 (14)	0.0273 (5)	
H7BA	−0.1052	0.4919	0.3294	0.033*	
H7BB	−0.0482	0.4177	0.3062	0.033*	
C7A	0.37489 (16)	0.53393 (13)	0.77222 (14)	0.0263 (5)	
H7AA	0.3804	0.4809	0.7946	0.032*	
H7AB	0.3130	0.5563	0.7855	0.032*	
C8A	0.46871 (15)	0.57758 (12)	0.82560 (13)	0.0222 (5)	
C8B	0.05145 (16)	0.50557 (12)	0.35655 (14)	0.0242 (5)	
C9A	0.47556 (16)	0.60980 (12)	0.91401 (14)	0.0229 (5)	
C9B	0.11666 (16)	0.46693 (13)	0.43265 (14)	0.0254 (5)	
C10A	0.38851 (16)	0.60200 (12)	0.95995 (13)	0.0230 (5)	
C10B	0.09194 (16)	0.38818 (13)	0.45760 (13)	0.0259 (5)	
C11A	0.34535 (17)	0.53268 (13)	0.97289 (14)	0.0276 (5)	
H11A	0.3696	0.4876	0.9509	0.033*	
C11B	−0.00181 (17)	0.36644 (14)	0.47148 (14)	0.0296 (5)	
H11B	−0.0561	0.4021	0.4625	0.036*	
C12B	−0.0166 (2)	0.29339 (15)	0.49820 (15)	0.0373 (6)	
H12A	−0.0808	0.2792	0.5080	0.045*	
C12A	0.26713 (18)	0.52905 (15)	1.01778 (15)	0.0344 (6)	
H12B	0.2379	0.4815	1.0263	0.041*	
C13A	0.23158 (18)	0.59423 (16)	1.05003 (16)	0.0389 (6)	
H13A	0.1767	0.5917	1.0791	0.047*	
C13B	0.0618 (2)	0.24065 (15)	0.51081 (15)	0.0407 (6)	
H13B	0.0510	0.1903	0.5286	0.049*	
C14A	0.27549 (19)	0.66288 (15)	1.04015 (16)	0.0374 (6)	
H14A	0.2519	0.7076	1.0635	0.045*	
C14B	0.1552 (2)	0.26133 (14)	0.49747 (15)	0.0374 (6)	
H14B	0.2090	0.2253	0.5059	0.045*	
C15A	0.35421 (17)	0.66683 (13)	0.99604 (15)	0.0297 (5)	
H15A	0.3851	0.7143	0.9904	0.036*	
C15B	0.17047 (18)	0.33420 (14)	0.47193 (14)	0.0326 (6)	
H15B	0.2355	0.3483	0.4638	0.039*	
C16B	−0.16076 (16)	0.61908 (13)	0.20724 (14)	0.0283 (5)	
C16A	0.48366 (18)	0.42951 (14)	0.65825 (17)	0.0361 (6)	
C17B	−0.18188 (19)	0.64543 (15)	0.28504 (16)	0.0383 (6)	
H17A	−0.1372	0.6460	0.3466	0.046*	
C18B	−0.28476 (18)	0.67257 (14)	0.25721 (16)	0.0349 (6)	
H18A	−0.3220	0.6948	0.2968	0.042*	
C19B	−0.31867 (17)	0.66084 (13)	0.16511 (16)	0.0306 (5)	
H19A	−0.3853	0.6736	0.1282	0.037*	
C17A	0.5364 (2)	0.38770 (16)	0.7306 (2)	0.0510 (7)	
H17B	0.5708	0.4048	0.7917	0.061*	
C18A	0.5296 (3)	0.31100 (17)	0.6957 (3)	0.0708 (10)	
H18B	0.5592	0.2674	0.7298	0.085*	
C19A	0.4745 (3)	0.31298 (19)	0.6076 (3)	0.0704 (10)	
H19B	0.4578	0.2700	0.5678	0.085*	
O4A	0.44496 (14)	0.38476 (11)	0.58157 (13)	0.0528 (5)	

### Atomic displacement parameters (Å^2^)

**Table:**

	*U*^11^	*U*^22^	*U*^33^	*U*^12^	*U*^13^	*U*^23^
O1A	0.0272 (8)	0.0292 (9)	0.0296 (8)	−0.0041 (7)	0.0045 (7)	−0.0069 (7)
O1B	0.0290 (9)	0.0367 (10)	0.0320 (8)	−0.0037 (7)	−0.0038 (7)	0.0021 (7)
O2A	0.0616 (13)	0.0578 (14)	0.0649 (13)	0.0333 (11)	0.0232 (11)	0.0109 (10)
O2B	0.0504 (12)	0.0714 (14)	0.0510 (11)	0.0082 (10)	0.0315 (10)	0.0077 (10)
O3A	0.0639 (13)	0.0571 (14)	0.0707 (13)	0.0140 (11)	0.0282 (11)	0.0324 (11)
O3B	0.0762 (15)	0.0410 (13)	0.0757 (14)	0.0046 (11)	0.0363 (12)	−0.0140 (11)
O4B	0.0270 (8)	0.0316 (9)	0.0241 (7)	0.0015 (7)	0.0007 (6)	0.0006 (7)
N1A	0.0256 (10)	0.0307 (11)	0.0349 (10)	−0.0038 (9)	0.0113 (8)	−0.0101 (9)
N1B	0.0257 (10)	0.0325 (11)	0.0351 (10)	−0.0060 (9)	0.0011 (8)	0.0024 (9)
N2A	0.0262 (10)	0.0343 (11)	0.0245 (9)	−0.0009 (8)	0.0102 (8)	−0.0028 (8)
N2B	0.0281 (10)	0.0340 (12)	0.0314 (10)	−0.0050 (9)	0.0020 (8)	0.0080 (9)
N3B	0.0303 (11)	0.0486 (15)	0.0300 (11)	0.0036 (10)	0.0026 (9)	−0.0072 (10)
N3A	0.0348 (11)	0.0368 (12)	0.0326 (10)	0.0032 (10)	0.0023 (9)	0.0030 (9)
C1A	0.0286 (13)	0.0381 (15)	0.0529 (15)	−0.0066 (11)	0.0189 (11)	−0.0051 (12)
C1B	0.0331 (15)	0.0450 (17)	0.0668 (19)	−0.0107 (13)	0.0059 (13)	0.0096 (14)
C3B	0.0450 (17)	0.0434 (18)	0.0596 (17)	−0.0068 (14)	0.0028 (14)	0.0196 (14)
C2B	0.071 (7)	0.055 (7)	0.036 (5)	−0.028 (5)	0.023 (5)	−0.002 (5)
C2D	0.046 (3)	0.035 (2)	0.035 (3)	−0.0075 (19)	0.0113 (19)	0.0070 (18)
C2A	0.0358 (14)	0.0585 (18)	0.0459 (15)	0.0001 (13)	0.0196 (12)	−0.0038 (13)
C3A	0.0356 (14)	0.0571 (18)	0.0384 (13)	−0.0031 (13)	0.0210 (11)	−0.0027 (12)
C4A	0.0235 (11)	0.0191 (11)	0.0267 (11)	0.0025 (9)	0.0052 (9)	0.0002 (9)
C4B	0.0213 (11)	0.0312 (13)	0.0232 (10)	0.0022 (10)	0.0062 (9)	−0.0005 (9)
C5A	0.0307 (12)	0.0342 (14)	0.0209 (10)	0.0016 (10)	0.0060 (9)	−0.0029 (9)
C5B	0.0258 (12)	0.0405 (15)	0.0209 (10)	−0.0006 (11)	0.0019 (9)	0.0043 (10)
C6A	0.0256 (11)	0.0271 (13)	0.0260 (11)	−0.0011 (10)	0.0036 (9)	−0.0023 (9)
C6B	0.0202 (11)	0.0378 (14)	0.0248 (11)	0.0030 (10)	0.0024 (9)	−0.0013 (10)
C7B	0.0244 (11)	0.0297 (13)	0.0273 (11)	−0.0004 (10)	0.0057 (9)	0.0000 (9)
C7A	0.0262 (12)	0.0284 (13)	0.0248 (11)	−0.0020 (10)	0.0076 (9)	−0.0025 (9)
C8A	0.0219 (11)	0.0216 (11)	0.0233 (10)	0.0026 (9)	0.0060 (9)	0.0004 (9)
C8B	0.0228 (11)	0.0270 (12)	0.0220 (10)	0.0006 (9)	0.0047 (9)	−0.0025 (9)
C9A	0.0242 (11)	0.0182 (11)	0.0248 (10)	0.0035 (9)	0.0035 (9)	0.0022 (9)
C9B	0.0248 (12)	0.0294 (13)	0.0217 (10)	0.0008 (10)	0.0055 (9)	−0.0037 (9)
C10A	0.0250 (11)	0.0235 (12)	0.0185 (10)	0.0022 (9)	0.0023 (9)	0.0017 (8)
C10B	0.0283 (12)	0.0318 (13)	0.0171 (10)	0.0001 (10)	0.0048 (9)	−0.0023 (9)
C11A	0.0340 (13)	0.0257 (13)	0.0225 (10)	0.0009 (10)	0.0061 (9)	−0.0002 (9)
C11B	0.0309 (12)	0.0363 (14)	0.0215 (11)	−0.0003 (10)	0.0064 (9)	−0.0006 (9)
C12B	0.0423 (15)	0.0474 (17)	0.0230 (11)	−0.0141 (13)	0.0100 (11)	−0.0005 (11)
C12A	0.0386 (14)	0.0383 (15)	0.0266 (11)	−0.0087 (12)	0.0091 (10)	0.0033 (10)
C13A	0.0338 (14)	0.0543 (18)	0.0332 (13)	0.0001 (12)	0.0174 (11)	0.0029 (12)
C13B	0.0623 (18)	0.0315 (15)	0.0256 (12)	−0.0072 (13)	0.0069 (12)	0.0028 (10)
C14A	0.0400 (14)	0.0393 (15)	0.0373 (13)	0.0111 (12)	0.0178 (11)	−0.0010 (11)
C14B	0.0503 (16)	0.0336 (15)	0.0273 (12)	0.0080 (12)	0.0083 (11)	0.0014 (10)
C15A	0.0354 (13)	0.0236 (12)	0.0302 (11)	0.0044 (10)	0.0087 (10)	0.0029 (10)
C15B	0.0333 (13)	0.0396 (15)	0.0255 (11)	0.0063 (11)	0.0087 (10)	0.0036 (10)
C16B	0.0264 (12)	0.0308 (13)	0.0246 (11)	−0.0019 (10)	0.0009 (9)	0.0023 (9)
C16A	0.0296 (13)	0.0353 (15)	0.0448 (14)	0.0008 (11)	0.0121 (11)	−0.0086 (12)
C17B	0.0367 (14)	0.0499 (17)	0.0244 (12)	0.0010 (12)	0.0008 (10)	−0.0051 (11)
C18B	0.0345 (14)	0.0344 (14)	0.0365 (13)	0.0008 (11)	0.0106 (11)	−0.0066 (11)
C19B	0.0230 (12)	0.0299 (13)	0.0368 (12)	0.0020 (10)	0.0043 (10)	0.0004 (10)
C17A	0.0348 (14)	0.0386 (17)	0.078 (2)	0.0049 (13)	0.0114 (14)	0.0096 (14)
C18A	0.0468 (19)	0.0324 (18)	0.145 (3)	0.0115 (14)	0.047 (2)	0.014 (2)
C19A	0.057 (2)	0.041 (2)	0.127 (3)	−0.0036 (16)	0.049 (2)	−0.027 (2)
O4A	0.0515 (12)	0.0452 (13)	0.0658 (12)	−0.0053 (10)	0.0224 (10)	−0.0292 (10)

### Geometric parameters (Å, º)

**Table:**

O1A—C9A	1.272 (2)	C6A—C7A	1.515 (3)
O1B—C9B	1.279 (3)	C6A—H6AA	1.0000
O2A—N3A	1.215 (3)	C6B—C7B	1.522 (3)
O2B—N3B	1.216 (3)	C6B—H6BA	1.0000
O3A—N3A	1.210 (3)	C7B—C8B	1.511 (3)
O3B—N3B	1.215 (3)	C7B—H7BA	0.9900
O4B—C16B	1.372 (2)	C7B—H7BB	0.9900
O4B—C19B	1.372 (3)	C7A—C8A	1.517 (3)
N1A—C4A	1.331 (3)	C7A—H7AA	0.9900
N1A—C1A	1.455 (3)	C7A—H7AB	0.9900
N1A—H1AA	0.8800	C8A—C9A	1.404 (3)
N1B—C4B	1.332 (3)	C8B—C9B	1.410 (3)
N1B—C1B	1.448 (3)	C9A—C10A	1.509 (3)
N1B—H1BA	0.8800	C9B—C10B	1.499 (3)
N2A—C4A	1.359 (3)	C10A—C11A	1.391 (3)
N2A—C5A	1.455 (3)	C10A—C15A	1.393 (3)
N2A—C3A	1.462 (3)	C10B—C11B	1.389 (3)
N2B—C4B	1.356 (3)	C10B—C15B	1.401 (3)
N2B—C3B	1.463 (3)	C11A—C12A	1.387 (3)
N2B—C5B	1.467 (3)	C11A—H11A	0.9500
N3B—C6B	1.519 (3)	C11B—C12B	1.379 (3)
N3A—C6A	1.525 (3)	C11B—H11B	0.9500
C1A—C2A	1.512 (4)	C12B—C13B	1.386 (4)
C1A—H1AB	0.9900	C12B—H12A	0.9500
C1A—H1AC	0.9900	C12A—C13A	1.380 (4)
C1B—C2B	1.385 (8)	C12A—H12B	0.9500
C1B—C2D	1.492 (5)	C13A—C14A	1.375 (4)
C1B—H1B1	0.9900	C13A—H13A	0.9500
C1B—H1B2	0.9900	C13B—C14B	1.376 (4)
C3B—C2D	1.451 (5)	C13B—H13B	0.9500
C3B—C2B	1.458 (10)	C14A—C15A	1.385 (3)
C3B—H3B1	0.9900	C14A—H14A	0.9500
C3B—H3B2	0.9900	C14B—C15B	1.373 (3)
C2B—H2B1	0.9900	C14B—H14B	0.9500
C2B—H2B2	0.9900	C15A—H15A	0.9500
C2D—H2DA	0.9900	C15B—H15B	0.9500
C2D—H2DB	0.9900	C16B—C17B	1.337 (3)
C2A—C3A	1.475 (4)	C16A—C17A	1.340 (4)
C2A—H2AA	0.9900	C16A—O4A	1.368 (3)
C2A—H2AB	0.9900	C17B—C18B	1.425 (3)
C3A—H3AA	0.9900	C17B—H17A	0.9500
C3A—H3AB	0.9900	C18B—C19B	1.332 (3)
C4A—C8A	1.427 (3)	C18B—H18A	0.9500
C4B—C8B	1.431 (3)	C19B—H19A	0.9500
C5A—C16A	1.487 (3)	C17A—C18A	1.445 (4)
C5A—C6A	1.523 (3)	C17A—H17B	0.9500
C5A—H5AA	1.0000	C18A—C19A	1.320 (5)
C5B—C16B	1.497 (3)	C18A—H18B	0.9500
C5B—C6B	1.520 (3)	C19A—O4A	1.355 (4)
C5B—H5BA	1.0000	C19A—H19B	0.9500
			
C16B—O4B—C19B	105.76 (16)	N3B—C6B—C5B	111.30 (19)
C4A—N1A—C1A	125.44 (19)	N3B—C6B—C7B	108.94 (18)
C4A—N1A—H1AA	117.3	C5B—C6B—C7B	111.72 (18)
C1A—N1A—H1AA	117.3	N3B—C6B—H6BA	108.3
C4B—N1B—C1B	124.6 (2)	C5B—C6B—H6BA	108.3
C4B—N1B—H1BA	117.7	C7B—C6B—H6BA	108.3
C1B—N1B—H1BA	117.7	C8B—C7B—C6B	111.35 (18)
C4A—N2A—C5A	123.06 (18)	C8B—C7B—H7BA	109.4
C4A—N2A—C3A	121.35 (19)	C6B—C7B—H7BA	109.4
C5A—N2A—C3A	115.33 (17)	C8B—C7B—H7BB	109.4
C4B—N2B—C3B	122.60 (19)	C6B—C7B—H7BB	109.4
C4B—N2B—C5B	123.62 (19)	H7BA—C7B—H7BB	108.0
C3B—N2B—C5B	113.75 (18)	C6A—C7A—C8A	112.21 (18)
O3B—N3B—O2B	123.7 (2)	C6A—C7A—H7AA	109.2
O3B—N3B—C6B	116.7 (2)	C8A—C7A—H7AA	109.2
O2B—N3B—C6B	119.7 (2)	C6A—C7A—H7AB	109.2
O3A—N3A—O2A	123.7 (2)	C8A—C7A—H7AB	109.2
O3A—N3A—C6A	119.0 (2)	H7AA—C7A—H7AB	107.9
O2A—N3A—C6A	117.2 (2)	C9A—C8A—C4A	119.54 (19)
N1A—C1A—C2A	109.4 (2)	C9A—C8A—C7A	122.16 (18)
N1A—C1A—H1AB	109.8	C4A—C8A—C7A	118.30 (17)
C2A—C1A—H1AB	109.8	C9B—C8B—C4B	119.84 (19)
N1A—C1A—H1AC	109.8	C9B—C8B—C7B	122.5 (2)
C2A—C1A—H1AC	109.8	C4B—C8B—C7B	117.59 (18)
H1AB—C1A—H1AC	108.2	O1A—C9A—C8A	125.0 (2)
C2B—C1B—N1B	116.0 (4)	O1A—C9A—C10A	114.30 (17)
N1B—C1B—C2D	110.0 (3)	C8A—C9A—C10A	120.67 (18)
C2B—C1B—H1B1	108.3	O1B—C9B—C8B	124.2 (2)
N1B—C1B—H1B1	108.3	O1B—C9B—C10B	114.91 (18)
C2D—C1B—H1B1	80.9	C8B—C9B—C10B	120.82 (19)
C2B—C1B—H1B2	108.3	C11A—C10A—C15A	118.6 (2)
N1B—C1B—H1B2	108.3	C11A—C10A—C9A	123.07 (19)
C2D—C1B—H1B2	135.6	C15A—C10A—C9A	118.2 (2)
H1B1—C1B—H1B2	107.4	C11B—C10B—C15B	118.2 (2)
C2D—C3B—N2B	114.4 (3)	C11B—C10B—C9B	124.3 (2)
C2B—C3B—N2B	113.4 (5)	C15B—C10B—C9B	117.4 (2)
C2D—C3B—H3B1	131.4	C12A—C11A—C10A	120.3 (2)
C2B—C3B—H3B1	108.9	C12A—C11A—H11A	119.8
N2B—C3B—H3B1	108.9	C10A—C11A—H11A	119.8
C2D—C3B—H3B2	78.4	C12B—C11B—C10B	120.4 (2)
C2B—C3B—H3B2	108.9	C12B—C11B—H11B	119.8
N2B—C3B—H3B2	108.9	C10B—C11B—H11B	119.8
H3B1—C3B—H3B2	107.7	C11B—C12B—C13B	120.4 (2)
C1B—C2B—C3B	119.0 (7)	C11B—C12B—H12A	119.8
C1B—C2B—H2B1	107.6	C13B—C12B—H12A	119.8
C3B—C2B—H2B1	107.6	C13A—C12A—C11A	120.2 (2)
C1B—C2B—H2B2	107.6	C13A—C12A—H12B	119.9
C3B—C2B—H2B2	107.6	C11A—C12A—H12B	119.9
H2B1—C2B—H2B2	107.0	C14A—C13A—C12A	120.1 (2)
C3B—C2D—C1B	112.7 (4)	C14A—C13A—H13A	119.9
C3B—C2D—H2DA	109.0	C12A—C13A—H13A	119.9
C1B—C2D—H2DA	109.0	C14B—C13B—C12B	119.9 (2)
C3B—C2D—H2DB	109.0	C14B—C13B—H13B	120.0
C1B—C2D—H2DB	109.0	C12B—C13B—H13B	120.0
H2DA—C2D—H2DB	107.8	C13A—C14A—C15A	120.0 (2)
C3A—C2A—C1A	110.1 (2)	C13A—C14A—H14A	120.0
C3A—C2A—H2AA	109.6	C15A—C14A—H14A	120.0
C1A—C2A—H2AA	109.6	C15B—C14B—C13B	119.8 (2)
C3A—C2A—H2AB	109.6	C15B—C14B—H14B	120.1
C1A—C2A—H2AB	109.6	C13B—C14B—H14B	120.1
H2AA—C2A—H2AB	108.2	C14A—C15A—C10A	120.7 (2)
N2A—C3A—C2A	111.2 (2)	C14A—C15A—H15A	119.6
N2A—C3A—H3AA	109.4	C10A—C15A—H15A	119.6
C2A—C3A—H3AA	109.4	C14B—C15B—C10B	121.2 (2)
N2A—C3A—H3AB	109.4	C14B—C15B—H15B	119.4
C2A—C3A—H3AB	109.4	C10B—C15B—H15B	119.4
H3AA—C3A—H3AB	108.0	C17B—C16B—O4B	110.6 (2)
N1A—C4A—N2A	118.30 (19)	C17B—C16B—C5B	133.6 (2)
N1A—C4A—C8A	120.18 (18)	O4B—C16B—C5B	115.78 (18)
N2A—C4A—C8A	121.52 (18)	C17A—C16A—O4A	110.4 (2)
N1B—C4B—N2B	118.0 (2)	C17A—C16A—C5A	134.0 (2)
N1B—C4B—C8B	120.21 (19)	O4A—C16A—C5A	115.6 (2)
N2B—C4B—C8B	121.77 (19)	C16B—C17B—C18B	106.3 (2)
N2A—C5A—C16A	110.09 (18)	C16B—C17B—H17A	126.9
N2A—C5A—C6A	111.40 (17)	C18B—C17B—H17A	126.9
C16A—C5A—C6A	112.28 (19)	C19B—C18B—C17B	106.9 (2)
N2A—C5A—H5AA	107.6	C19B—C18B—H18A	126.5
C16A—C5A—H5AA	107.6	C17B—C18B—H18A	126.5
C6A—C5A—H5AA	107.6	C18B—C19B—O4B	110.4 (2)
N2B—C5B—C16B	110.12 (19)	C18B—C19B—H19A	124.8
N2B—C5B—C6B	111.65 (18)	O4B—C19B—H19A	124.8
C16B—C5B—C6B	109.94 (19)	C16A—C17A—C18A	105.2 (3)
N2B—C5B—H5BA	108.3	C16A—C17A—H17B	127.4
C16B—C5B—H5BA	108.3	C18A—C17A—H17B	127.4
C6B—C5B—H5BA	108.3	C19A—C18A—C17A	107.1 (3)
C7A—C6A—C5A	111.15 (17)	C19A—C18A—H18B	126.5
C7A—C6A—N3A	110.20 (18)	C17A—C18A—H18B	126.5
C5A—C6A—N3A	110.05 (18)	C18A—C19A—O4A	110.6 (3)
C7A—C6A—H6AA	108.5	C18A—C19A—H19B	124.7
C5A—C6A—H6AA	108.5	O4A—C19A—H19B	124.7
N3A—C6A—H6AA	108.5	C19A—O4A—C16A	106.7 (3)
			
C4A—N1A—C1A—C2A	−18.9 (3)	C6A—C7A—C8A—C9A	154.7 (2)
C4B—N1B—C1B—C2B	−3.5 (10)	C6A—C7A—C8A—C4A	−26.1 (3)
C4B—N1B—C1B—C2D	31.7 (4)	N1B—C4B—C8B—C9B	−2.6 (3)
C4B—N2B—C3B—C2D	−14.8 (5)	N2B—C4B—C8B—C9B	175.7 (2)
C5B—N2B—C3B—C2D	167.2 (4)	N1B—C4B—C8B—C7B	179.52 (19)
C4B—N2B—C3B—C2B	21.1 (9)	N2B—C4B—C8B—C7B	−2.2 (3)
C5B—N2B—C3B—C2B	−157.0 (8)	C6B—C7B—C8B—C9B	−146.8 (2)
N1B—C1B—C2B—C3B	22.3 (18)	C6B—C7B—C8B—C4B	31.0 (3)
C2D—C1B—C2B—C3B	−64.8 (12)	C4A—C8A—C9A—O1A	1.8 (3)
C2D—C3B—C2B—C1B	68.9 (11)	C7A—C8A—C9A—O1A	−178.90 (19)
N2B—C3B—C2B—C1B	−30.2 (17)	C4A—C8A—C9A—C10A	−179.19 (18)
C2B—C3B—C2D—C1B	−55.2 (7)	C7A—C8A—C9A—C10A	0.1 (3)
N2B—C3B—C2D—C1B	40.6 (6)	C4B—C8B—C9B—O1B	4.1 (3)
C2B—C1B—C2D—C3B	59.6 (7)	C7B—C8B—C9B—O1B	−178.2 (2)
N1B—C1B—C2D—C3B	−47.5 (6)	C4B—C8B—C9B—C10B	−172.25 (19)
N1A—C1A—C2A—C3A	49.2 (3)	C7B—C8B—C9B—C10B	5.5 (3)
C4A—N2A—C3A—C2A	28.4 (3)	O1A—C9A—C10A—C11A	−128.2 (2)
C5A—N2A—C3A—C2A	−145.9 (2)	C8A—C9A—C10A—C11A	52.7 (3)
C1A—C2A—C3A—N2A	−54.2 (3)	O1A—C9A—C10A—C15A	47.8 (3)
C1A—N1A—C4A—N2A	−8.3 (3)	C8A—C9A—C10A—C15A	−131.3 (2)
C1A—N1A—C4A—C8A	172.0 (2)	O1B—C9B—C10B—C11B	131.8 (2)
C5A—N2A—C4A—N1A	177.48 (19)	C8B—C9B—C10B—C11B	−51.6 (3)
C3A—N2A—C4A—N1A	3.6 (3)	O1B—C9B—C10B—C15B	−45.1 (3)
C5A—N2A—C4A—C8A	−2.9 (3)	C8B—C9B—C10B—C15B	131.5 (2)
C3A—N2A—C4A—C8A	−176.8 (2)	C15A—C10A—C11A—C12A	2.4 (3)
C1B—N1B—C4B—N2B	−5.9 (3)	C9A—C10A—C11A—C12A	178.36 (18)
C1B—N1B—C4B—C8B	172.5 (2)	C15B—C10B—C11B—C12B	−0.3 (3)
C3B—N2B—C4B—N1B	−3.8 (3)	C9B—C10B—C11B—C12B	−177.24 (19)
C5B—N2B—C4B—N1B	174.1 (2)	C10B—C11B—C12B—C13B	−0.5 (3)
C3B—N2B—C4B—C8B	177.9 (2)	C10A—C11A—C12A—C13A	−0.1 (3)
C5B—N2B—C4B—C8B	−4.2 (3)	C11A—C12A—C13A—C14A	−1.8 (3)
C4A—N2A—C5A—C16A	−97.1 (2)	C11B—C12B—C13B—C14B	0.6 (3)
C3A—N2A—C5A—C16A	77.1 (2)	C12A—C13A—C14A—C15A	1.3 (4)
C4A—N2A—C5A—C6A	28.1 (3)	C12B—C13B—C14B—C15B	0.2 (3)
C3A—N2A—C5A—C6A	−157.7 (2)	C13A—C14A—C15A—C10A	1.1 (3)
C4B—N2B—C5B—C16B	103.4 (2)	C11A—C10A—C15A—C14A	−2.9 (3)
C3B—N2B—C5B—C16B	−78.5 (2)	C9A—C10A—C15A—C14A	−179.09 (19)
C4B—N2B—C5B—C6B	−19.0 (3)	C13B—C14B—C15B—C10B	−1.0 (3)
C3B—N2B—C5B—C6B	159.1 (2)	C11B—C10B—C15B—C14B	1.1 (3)
N2A—C5A—C6A—C7A	−51.2 (2)	C9B—C10B—C15B—C14B	178.21 (19)
C16A—C5A—C6A—C7A	72.8 (2)	C19B—O4B—C16B—C17B	−0.1 (3)
N2A—C5A—C6A—N3A	71.2 (2)	C19B—O4B—C16B—C5B	178.80 (19)
C16A—C5A—C6A—N3A	−164.79 (18)	N2B—C5B—C16B—C17B	−34.3 (4)
O3A—N3A—C6A—C7A	139.3 (2)	C6B—C5B—C16B—C17B	89.1 (3)
O2A—N3A—C6A—C7A	−44.7 (3)	N2B—C5B—C16B—O4B	147.02 (18)
O3A—N3A—C6A—C5A	16.4 (3)	C6B—C5B—C16B—O4B	−89.5 (2)
O2A—N3A—C6A—C5A	−167.7 (2)	N2A—C5A—C16A—C17A	32.9 (4)
O3B—N3B—C6B—C5B	176.92 (19)	C6A—C5A—C16A—C17A	−91.8 (3)
O2B—N3B—C6B—C5B	−3.7 (3)	N2A—C5A—C16A—O4A	−144.83 (19)
O3B—N3B—C6B—C7B	53.3 (3)	C6A—C5A—C16A—O4A	90.4 (2)
O2B—N3B—C6B—C7B	−127.3 (2)	O4B—C16B—C17B—C18B	0.1 (3)
N2B—C5B—C6B—N3B	−74.8 (2)	C5B—C16B—C17B—C18B	−178.6 (2)
C16B—C5B—C6B—N3B	162.72 (16)	C16B—C17B—C18B—C19B	0.0 (3)
N2B—C5B—C6B—C7B	47.3 (2)	C17B—C18B—C19B—O4B	−0.1 (3)
C16B—C5B—C6B—C7B	−75.3 (2)	C16B—O4B—C19B—C18B	0.2 (3)
N3B—C6B—C7B—C8B	69.9 (2)	O4A—C16A—C17A—C18A	0.0 (3)
C5B—C6B—C7B—C8B	−53.4 (2)	C5A—C16A—C17A—C18A	−177.8 (3)
C5A—C6A—C7A—C8A	50.4 (2)	C16A—C17A—C18A—C19A	−0.2 (3)
N3A—C6A—C7A—C8A	−71.9 (2)	C17A—C18A—C19A—O4A	0.3 (4)
N1A—C4A—C8A—C9A	0.5 (3)	C18A—C19A—O4A—C16A	−0.3 (3)
N2A—C4A—C8A—C9A	−179.12 (19)	C17A—C16A—O4A—C19A	0.2 (3)
N1A—C4A—C8A—C7A	−178.80 (19)	C5A—C16A—O4A—C19A	178.5 (2)
N2A—C4A—C8A—C7A	1.6 (3)		

### Hydrogen-bond geometry (Å, º)

**Table:**

*D*—H···*A*	*D*—H	H···*A*	*D*···*A*	*D*—H···*A*
N1*A*—H1*AA*···O1*A*	0.88	1.84	2.553 (2)	137
N1*B*—H1*BA*···O1*B*	0.88	1.85	2.556 (3)	136
C6*A*—H6*AA*···O1*B*	1.00	2.36	3.103 (3)	130
C7*A*—H7*AB*···O2*A*	0.99	2.44	2.790 (3)	100

## References

[bb1] Higashi, T. (1995). *ABSCOR* Rigaku Corporation, Tokyo, Japan.

[bb2] Huang, Z.-T. & Wang, M.-X. (1994). *Heterocycles*, **37**, 1233–1262.

[bb3] Rigaku (2001). *R*APID-AUTO. Rigaku Corporation, Tokyo, Japan.

[bb4] Sheldrick, G. M. (2008). *Acta Cryst.* A**64**, 112–122. 10.1107/S0108767307043930 18156677

[bb5] Yaqub, M., Yu, C.-Y., Jia, Y.-M. & Huang, Z.-T. (2008). *Synlett*, **9**, 1357–1360.

[bb6] Yu, C.-Y., Yang, P.-H., Zhao, M.-X. & Huang, Z.-T. (2006). *Synlett*, pp. 1835–1840.

[bb7] Yu, C.-Y., Yuan, X.-N. & Huang, Z.-T. (2007). *Acta Cryst.* E**63**, o3186.

